# National Insurance and the Proposed Ministry of Health

**Published:** 1917-05-12

**Authors:** 


### NATIONAL INSURANCE ACT DEVELOPMENTS.

### National Insurance and the Proposed Ministry of Health.

The knowledge that the question of setting up a Ministry of Health is now receiving the definite consideration of the Government, and that the introduction of a Bill to give effect to the matter is impending has created quite a stir among approved society officials and the various organisations in which many approved societies are associated. Insurance Committees also are actively concerning themselves in the matter, as indicated by the report published in The Hospital for April 21 (page 46) of the interview accorded to a deputation, representing the National Associations of Insurance Committees for England and Wales, by Lord Rhondda at the offices of the Local Government Board on April 13. But it is misleading to suppose that the opinions expressed by that deputation in any way represent the considered judgment of National Health Insurance societies as a whole. The Associations of Insurance Committees do not, and cannot in any sense, represent approved societies, although it appears that some such claim was put forward by the deputation. Indeed, it would even seem that the Associations do not truly represent the opinion of Insurance Committees as a whole, for several of the Committees—including the London Insurance Committee—have not identified themselves with the Association. The overtures made by this deputation have, therefore, caused considerable annoyance to the leaders of the principal approved societies, and they are taking steps to rectify any misconception to which the interview may have given rise.

### Approved Societies' Protest.

It is not that the leaders in the approved society world object on principle to the institution of a Ministry of Health—on the contrary, they appear to welcome the idea—but it is clear that they will not be content to allow the administration of approved societies to come under the direct ægis of the Local Government Board without making a strenuous resistance. The attitude of the approved societies is expressed very emphatically in a leading article—evidently inspired by important society officials—which appeared in the *National Insurance Gazette* for April 7, and from which the following is an extract:—

Our present purpose is to tell approved societies and National Insurance workers in general, how very serious is the situation, and how very much they need to be on the alert to check any encroachments upon their legitimate work by Lord Rhondda's scheme. We earnestly call upon them to consider the situation, to hold the necessary meetings, and attend all conferences possible, so that the leaders in the National Insurance world may be able to approach the Government with authority. Without speaking in any alarmist manner we would say that the answer to the present question will decide very largely the fate of insurance committees, and may, indeed, imperil approved societies, friendly societies, and other institutions working the National Insurance Acts. Co-ordination is a good word, but it means pushing somebody out in too many instances. In the present instance, if we are not very careful indeed, we shall be amongst those pushed out, or at least pushed into the background. We trust that every National Insurance worker in the kingdom will realise this, and will do his best, either by attending meetings or otherwise, to put up a good fight. It is really a very serious matter, and certainly the most menacing one that National Insurance has yet had to meet.

It had been intended to hold a mass meeting at the Central Hall, Westminster, on April 16 of all those who are interested in National Insurance administration to protest against the Local Government Board becoming in any way the central authority for the administration of National Health matters. Sir Edwin Cornwall, the present Insurance Minister, was to have been the principal speaker, but owing to his inability to attend, the meeting was at the last moment postponed. We have as yet, therefore, no definite knowledge of the grounds upon which the insurance workers base their objection to the present scheme. It is intended, however, to convene a large meeting early in May with the object of making the protest effectual.

### Why this Show of Fight?

Now, looking at the matter from an outside and detached point of view, without any prejudice in favour of or against approved societies, but with the sole purpose of securing the greatest advantage to public health, one is forced to consider if there are any substantial grounds for all this show of fight. Why is it that National Insurance workers are afraid that approved societies will be "pushed out"? Is it because they have not taken pains to secure proper information as to what is proceeding? Or do they resent the fact that the initiative has been taken by the President of the Local Government Board rather than by the Insurance Minister? If National Insurance workers have been slow to press upon the heads of their own department of National Health administration the necessity and urgency for the co-ordination of all the overlapping authorities at present in charge of public health, they have only themselves to blame. If they have been pressing for this reform without securing an effective response from their own officials, they should welcome the prospect of the fulfilment of their desires, even though the initiative comes from another Government Department.

We fear, however, that approved society officials and National Insurance workers generally have been too much immersed in their own affairs—owing to the acknowledged intricacies of the cumbrous system under which they have had to work—to give much attention to the larger problem that now confronts them. They would, doubtless, have wakened to the necessity of an extension of health protective measures in the course of a comparatively short time. There are already evidences of this in the Final Report of the Departmental

Inquiry, in which, among other things, the Committee drew special attention to the importance of the housing problem as affecting the treatment of tuberculous patients, and the stamping out of the white plague. It is not necessarily the fault either of the officials of approved societies, or of Insurance Committees, or even of the Insurance Commissioners, that they have not already taken steps to secure the co-ordination of the Health Services of the nation. National Insurance is comparatively new—we are apt to forget that it is not yet five years since the Act of 1911 came into operation—and ideas on the larger aspect of the work which they have had to undertake have had to germinate as well as grow in the minds of its recognised administrators. Their task has not been an easy one—it has been hampered, unfortunately, by political animosities and jealousies—but, in spite of difficulties, it can be said that National Insurance has so far justified its inception that any scheme for centralising the National Health Services under a Ministry of Health could not possibly ignore the very valuable machinery that is ready to hand in the approved societies, Insurance Committees, and the Insurance Commissions.

### The Need for a Mutual Understanding.

Why, then, is there this feeling of uneasiness on the part of approved society officials? Is it that they are standing aloof, desirous of securing some ends of their own—what, we know not—when they ought to be co-operating with Lord Rhondda as representing the Government, seeing that the Local Government Board must be outside the proposed Ministry of Health, for this Ministry must be free from all taint and connection with the Poor Law. To succeed this Ministry must be independent, and the most effective organisation for the benefit of the health of the community that can be devised. There should first be a mutual understanding between all parties concerned. Exact information as to the present intentions of Lord Rhondda and those who are collaborating with him should be made available at the earliest possible moment. Only by such means can unfounded rumours be effectively disposed of, and harmony secured sufficiently strong to bring to the cause a noble army of workers ready to criticise details, if need be, but not to obstruct or oppose the general scheme just because individual interests may in some instances be affected.

### Lord Rhondda's Desire for Co-operation.

Although Lord Rhondda made it clear that he was only expressing his own private opinion, he also made it evident that he was pressing his views upon the Government. It is, therefore, very significant that he should have said "with regard to the position that, in my opinion, should be occupied by the Health Insurance Committees under that central authority, I am sure I do not know where the rumours you have referred to were got from, but I have never had any thought or intention, and I have never considered it really from the point of view, of abolishing the Health Insurance Committees. I want to work with their full co-operation; I want to get all the assistance I can from them. I wish you to understand that thoroughly. With regard to approved societies, whenever the housing question and the establishment of a Ministry of Health are taken in hand, and I hope it will be very soon, I hope they will be taken in hand on strictly non-party lines, not one party trying for a moment to get the better of the other. . . . I should not be a very far-seeing man if I failed to realise that I should not secure practical results in a reasonable time if I set myself up against the general medical practitioner and against the approved societies. I want to work with the fullest co-operation of all those who are now interested in health matters, and not to displace them or to restrict their operations or restrict their development."

### The Outlines of a Practical Scheme.

It may be that for some while to come the centralisation of health administration under one comprehensive Ministry of Health will prove too gigantic an undertaking to be given practical effect. But that is no reason why the initial steps should not be taken in the direction of securing this as the ultimate ideal. At the present time, as shown by the excellent report recently issued by a small Committee consisting of members of Parliament, of which Major the Hon. Waldorf Astor was chairman, under the title "The Health of the People—A New National Policy,"* although there are six separate and independent Departments of State, which deal incidentally with public health as an accessory to their more characteristic duties, there is no single Department of which health constitutes the primary function and *raison d'être*. This Committee have already done much in bringing within a small compass a comprehensive statement of the complex and overlapping character of the present public health administration. They go further, however, and recommend that the reorganisation scheme should produce a single national authority pre-eminently concerned with health. This authority, they suggest, should absorb forthwith the health activities of the Local Government Board and the Insurance Department, co-ordinating them and giving them free scope for development. They do not propose, however, that the remaining Departments of State which are at present in charge of various sectional aspects of public health, should be immediately divested of their powers in regard thereto. The solution put forward is that of appointing an Advisory Committee, on which the head medical officers of all such Departments should be represented, to act in co-operation with the Health Department.

When instituting a new central authority, Parliament must secure that all taint of pauperism shall be eliminated. It is the possibility of such taint which National Insurance workers fear in countenancing in any way the amalgamation of National Insurance with Local Government Board administration. Lord Rhondda is himself sensitive

* Argus Printing Company, Ltd., 10 Temple Avenue, E.C. 4, price 3d.

on the point, and has expressed the desirability of entirely removing such taint from the activities of the central Health Department, whatever authority it may be.

Here, then, is a field for useful national enterprise in which National Insurance workers can join just as heartily as anyone else. At present there is waste of effort both centrally and locally. A comprehensive scheme of reform must be undertaken. The problem becomes more urgent day by day and week by week. Let National Insurance workers rise to their opportunity and work with a will, not for obstruction, but by hearty co-operation with Lord Rhondda for the institution of a standard of public health administration that shall be the envy of the world, and that shall bear fruit in increased prosperity, happiness, and all that tends to the well-being of the community as a whole.

